# Myosin II ATPase Activity Mediates the Long-Term Potentiation-Induced Exodus of Stable F-Actin Bound by Drebrin A from Dendritic Spines

**DOI:** 10.1371/journal.pone.0085367

**Published:** 2014-01-22

**Authors:** Toshiyuki Mizui, Yuko Sekino, Hiroyuki Yamazaki, Yuta Ishizuka, Hideto Takahashi, Nobuhiko Kojima, Masami Kojima, Tomoaki Shirao

**Affiliations:** 1 Department of Neurobiology and Behavior, Gunma University Graduate School of Medicine, Maebashi, Gunma, Japan; 2 Core Research for Evolution Science and Technology, Japan Science and Technology Corporation, Kawaguchi, Saitama, Japan; 3 Bio-interface Research Group, Health Research Institute, National Institute of Advanced Industrial Science and Technology (AIST), Ikeda, Osaka, Japan; Osaka University Graduate School of Medicine, Japan

## Abstract

The neuronal actin-binding protein drebrin A forms a stable structure with F-actin in dendritic spines. NMDA receptor activation causes an exodus of F-actin bound by drebrin A (DA-actin) from dendritic spines, suggesting a pivotal role for DA-actin exodus in synaptic plasticity. We quantitatively assessed the extent of DA-actin localization to spines using the spine-dendrite ratio of drebrin A in cultured hippocampal neurons, and found that (1) chemical long-term potentiation (LTP) stimulation induces rapid DA-actin exodus and subsequent DA-actin re-entry in dendritic spines, (2) Ca^2+^ influx through NMDA receptors regulates the exodus and the basal accumulation of DA-actin, and (3) the DA-actin exodus is blocked by myosin II ATPase inhibitor, but is not blocked by myosin light chain kinase (MLCK) or Rho-associated kinase (ROCK) inhibitors. These results indicate that myosin II mediates the interaction between NMDA receptor activation and DA-actin exodus in LTP induction. Furthermore, myosin II seems to be activated by a rapid actin-linked mechanism rather than slow MLC phosphorylation. Thus the myosin-II mediated DA-actin exodus might be an initial event in LTP induction, triggering actin polymerization and spine enlargement.

## Introduction

Drebrin A is a neuron-specific actin-binding protein that is located at the base of dendritic spine heads [1]–[3]. Drebrin A binding modifies the pitch of actin filaments [4]–[5] and forms stable F-actin that is resistant to depolymerization by cytochalasin D [6]–[7]. Mikati *et al* [8] have recently shown that F-actin that is bound by drebrin A (DA-actin) is stable, and the depolymerization of DA-actin is suppressed at both ends of the filaments. In developing neurons, DA-actin is not observed in dynamic dendritic filopodia, but is observed in more stable dendritic spines [9]. Furthermore, DA-actin is suitable for the formation of a stable higher-order F-actin structure (DA-actin complex), because drebrin A has two F-actin-binding domains that can act cooperatively to enable interfilament interactions [10]. Together, it is suggested that DA-actin forms stable structures of F-actin at the base of dendritic spine heads.

Dendritic spines at rest contain a dynamic F-actin pool that shows quick treadmilling, and a stable pool that shows slow treadmilling. The dynamic pool is observed at the tip of the spine head, whereas the stable pool is located at the base of the spine head [11]. The aforementioned stability and localization of DA-actin suggest that DA-actin is a major component of the stable F-actin pool in dendritic spines, whereas F-actin that is not bound by drebrin A (non-DA-actin) is a major component of the dynamic F-actin pool (for review, see Shirao and González-Billault [12]).

We have previously shown that NMDA receptor activation induces the loss of drebrin A from dendritic spines [13]. Because our previous biochemical analyses revealed that most drebrin A in neurons is bound to F-actin (for a review, see Sekino *et al* [14]), we can extrapolate DA-actin localization from drebrin immunostaining images. Furthermore, fluorescence recovery after photobleaching analysis has demonstrated that drebrin A dynamics in spines are far slower than those of monomeric proteins such as monomeric actin [15] and cortactin [16], indicating that drebrin A remains bound to F-actin even when drebrin A dynamically changes its localization [17]. Together, it is suggested that NMDA receptor activation induces the DA-actin exodus from dendritic spines. However, the mechanism by which NMDA receptor activation links to the DA-actin exodus remains to be elucidated.

It has recently been reported that myosin II ATPase activity is necessary for actin reorganization during long-term potentiation (LTP) induction [18]. We have previously shown that myosin II is contained in the DA-actin complex [19], but the interaction between myosin II and DA-actin is suppressed in the DA-actin complex [20]. The release of the actomyosin interaction leads to the activation of myosin II ATPase, resulting in reorganization of the actin cytoskeleton through severing of F-actin [21]. Therefore, LTP stimulation might disinhibit the suppressed actomyosin interaction, resulting in the activation of myosin II ATPase.

In this study, we quantitatively assessed the extent to which DA-actin localizes to dendritic spines, clarified DA-actin migration in the context of synaptic plasticity and examined whether myosin II mediates the interaction between NMDA receptor activation and DA-actin exodus.

## Materials and Methods

All animal experiments were performed with the permission of the Animal Care and Experimentation Committee, Gunma University, Showa Campus (Maebashi, Japan). All efforts were made to minimize animal suffering and reduce the number of animals used in this study.

### Reagents

4-aminopyridine (4-AP) and pyruvate were purchased from Sigma (St. Louis, MO, USA). Hexahydro-1-[(5-iodo-1-naphthalenyl) sulfonyl]-1*H*-1,4-diazepine hydrochloride (ML-7) and (*S*)-(+)-2-Methyl-1-[(4-methyl-5-isoquinolinyl)sulfonyl]-hexahydro-1*H*-1,4-diazepine dihydrochloride (H-1152) were purchased from Calbiochem (San Diego, CA, USA). Ethyleneglycol-*bis* (β-aminoethyl)-N,N,N',N'-tetraacetic acid (EGTA) was purchased from Dojin (Kumamoto, Japan). Tetrodotoxin (TTX) was purchased from Wako (Osaka, Japan). D-(−)-2-amino-5-phosphonopentanoic acid (APV), bicuculline, (S)-(−)-blebbistatin, (R)-(+)-blebbistatin, thapsigargin and nifedipine were purchased from Tocris (Ellisville, MO, USA).

### Hippocampal neuron cultures

Timed pregnant Wistar rats (Charles River Laboratories Inc., Yokohama, Japan) were deeply anesthetized with diethyl ether and sacrificed by decapitation. Hippocampi were dissected from the fetuses at embryonic day 18. The hippocampal cells were prepared by trypsinization and mechanical dissociation according to previously described methods [9]. Briefly, the cell suspensions were plated at a density of 5000 cells/cm^2^ on coverslips coated with poly-L-lysine (Sigma). Cells were incubated in Minimum Essential Medium (MEM; Invitrogen, San Diego, CA, USA) supplemented with 10% fetal bovine serum (Invitrogen), 0.6% glucose (Wako), and 1 mM pyruvate (Sigma). After the cells achieved attachment, the coverslips were transferred to a culture dish containing a glial monolayer and maintained in normal medium consisting of serum-free MEM, 2% B27 supplement (Invitrogen), 0.6% glucose, and 1 mM sodium pyruvate at 35.8°C in a humidified incubator with 5% CO_2_ for 21 days *in vitro* (DIV). Cytosine β-D-arabinofuranoside (10 µM; Sigma) was added to the cultures at 4 DIV to inhibit glial proliferation.

### Pharmacological treatments

The chemical LTP (cLTP) stimulation solution used in this study was Mg^2+^-free Tyrode's solution supplemented with 200 µM glycine, 20 µM bicuculline, 1 µM strychnine and 0.5 µM TTX [22]. For cLTP induction, the neurons were preincubated in cLTP stimulation solution without 200 µM glycine for 20 min, and then stimulated with glycine for the indicated amount of time.

For blocker experiments, the neurons were preincubated in medium supplemented with blocker for 30 min and then stimulated with cLTP stimulation solution or 100 µM glutamate in the presence of each blocker for the indicated amount of time. For the stimulation studies with 90 mM potassium chloride, the neurons were preincubated in Tyrode's solution (119 mM NaCl, 2.5 mM KCl, 2 mM CaCl_2_, 2 mM MgSO_4_, 25 mM HEPES [pH 7.4], and 30 mM glucose).

### Immunocytochemistry

After three weeks *in vitro*, cells were fixed in 4% paraformaldehyde with 0.1% glutaraldehyde in phosphate-buffered saline (PBS; pH 7.4) at 4°C for 10 min. The fixed cells were permeabilized with 0.1% Triton X-100 in PBS for 3 min and then incubated in blocking solution (3% bovine serum albumin in PBS) for 1 h, followed by an overnight incubation with primary antibodies at 4°C. A monoclonal antibody against drebrin (clone M2F6, hybridoma supernatant [23]) was used at a 1∶1 dilution. F-actin was detected with rhodamine-conjugated phalloidin (Molecular Probes, Eugene, OR, USA). After being washed with PBS for 30 min, the cells were incubated for 1 h at room temperature with secondary antibodies. Anti-mouse IgG antibodies conjugated to fluorescein (Cappel, West Chester, PA, USA) were used to detect the monoclonal antibodies against drebrin. After being washed with PBS, the cells were mounted on glass slides with Perma Fluor mounting medium (Thermo Shandon, Pittsburgh, PA, USA).

Labeling of surface GluR1 was performed according to previous reports [22], [24] with minor modifications. Briefly, after the cLTP stimulation, surface GluR1 was labeled in live neurons by 30 min incubation with a rabbit polyclonal antibody against the N-terminus of the GluR1 subunit (PC246, 1∶15 dilution in glial conditioned media; Calbiochem). After washout of the antibody with Hank's balanced salt solution, the neurons were fixed in 2% PFA for 20 min at 4°C without permeabilization. The surface receptors were visualized using a fluorescein-conjugated goat anti-rabbit secondary antibody (Cappel).

### Fluorescence microscopy

All fluorescence images were obtained on a Zeiss Axioplan 2 microscope (Zeiss, Jena, Germany) equipped with a Cool Snap fx CCD camera (Photometrics, Tucson, AZ, USA), a 63×, 1.4 numerical aperture objective lens (Zeiss), and MetaMorph software (Universal Imaging, West Chester, PA, USA). A filter set (86000 Sedat Quad; Chroma, Brattleboro, VT, USA) was mounted in the excitation and emission filter wheels (Ludl Electronic Products, Hawthorne, NY, USA) of the microscope. All data were collected at 1300×1030 resolution at 12 bits/pixel. A single pixel in the image corresponded to a 106 nm^2^ area in the specimen plane. The images used for comparison in this study were collected under identical conditions. The captured fluorescence images were analyzed using the MetaMorph program. The GFP, rhodamine, and Cy5 signals were obtained through filters for FITC, Cy3, and Cy5, respectively. We found no fluorescence leakage of these signals through the other filters. The images presented in this study were prepared using Adobe Photoshop software (Adobe Systems, San Jose, CA, USA).

### Plasmids and Transfection

Construction of the green fluorescent protein-tagged drebrin A (GFP-DA) has been described previously [25]. At 7 DIV, the hippocampal neurons were transfected with plasmids using a calcium phosphate coprecipitation protocol [26]. Two weeks after transfection, the transfected neurons were analyzed using time-lapse imaging.

### Time-lapse imaging

Live time-lapse imaging was performed at 35.8°C on a Zeiss inverted microscope stage using a temperature-controlled chamber with continuous perfusion, as described previously [7]. Briefly, the cell cultures were mounted in a chamber containing Mg^2+^-free Tyrode's solution supplemented with 20 µM bicuculline, 1 µM strychnine and 0.5 µM TTX. The chamber was maintained and perfused with the same solution. The perfusing solution (1 ml/min) was switched between the control and cLTP stimulation medium using a hydraulic two-way valve switch with a dead space of 3.5 ml between the switch and the bath. Thus, the test medium would reach the chamber 210 sec after flipping the switch (data not shown). The time-lapse images were acquired at 10 sec intervals for 20 min using MetaMorph software.

### Image analysis and quantification

All quantifications were performed by an observer who was blind to the experimental conditions, and the morphological analysis was performed using the MetaMorph software. Each experiment was repeated at least three times with independent neuronal preparations.

For quantification, spiny neurons with pyramidal morphology were selected from at least three separate cultures. In our hippocampal cultures, we have previously shown that pyramidal neurons are multipolar cells with a large soma and multiple thick dendritic processes [27]. Therefore, we considered that these multipolar cells were hippocampal pyramidal neurons. Although the hippocampal culture also contains a variety of interneurons, they are comparatively few in number and most are morphologically distinguishable in culture [28].

To measure the surface cluster density of GluR1, the number of GluR1 clusters was measured according to previously described methods [24], [29] with minor modifications. Briefly, the surface GluR1 signals were thresholded with intensity at two-fold the dendritic background to mark surface clusters of GluR1 using MetaMorph software. The surface clusters were selected using the ‘regions’ tool and analyzed using the ‘integrated morphometry’ feature. Next, the length of the analyzed region on a dendrite was measured. The density of surface GluR1 clusters were obtained by dividing the number of surface GluR1 clusters within a selected dendritic region by the length of the dendrite (50–100 µm total dendritic length per neuron).

### Calculation of the spine-dendrite ratio

**Figure 1:**
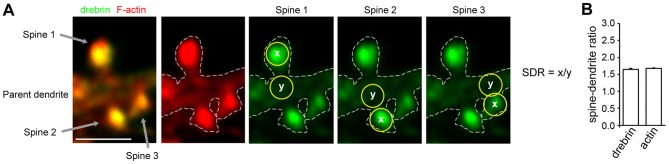
Spine-dendrite ratios of drebrin and actin. (**A**) Fluorescence images of drebrin (green) and F-actin (red) in control hippocampal neurons. The dendritic spines and the parent dendrite in the selected region were outlined on the F-actin fluorescence images (white dotted lines). Scale bar, 2 µm. Circles (0.26 µm^2^, yellow) were drawn at the spine head and at the parent dendrite, and the average fluorescence intensities within the circles were measured. The SDR of each spine was obtained by dividing the average intensity of the dendritic spine by that of the parent dendrite. (**B**) The SDR per neuron was obtained as the average SDR of the spines. Bar graph shows the drebrin and actin SDRs of 21-DIV hippocampal neurons (n = 170 cells). Error bars represent s.e.m.

The spine-dendrite ratio (SDR) used in this study was the average fluorescence signal of the molecule of interest in a dendritic spine head divided by the average fluorescence signal of that molecule in the dendritic shaft at the foot of the spine. To measure the SDR, a single dendrite located between the cell soma and the second branch point (30–80 µm total dendritic length per neuron) was selected from each neuron. The dendritic spines and the shaft in the selected region were outlined on the F-actin fluorescence images, and circles (0.26 µm^2^) were drawn at dendritic spine heads and at the foot of the spine in a dendritic shaft, using the ‘ellipse region’ tool of the MetaMorph software (Fig. 1A). Next, the average fluorescence intensity in each circle (calculated from the fluorescence signal intensity values) was measured using the ‘region measurements’ tool in the MetaMorph software. The number of spines measured were between 25 and 80 per neuron. The average SDR of spines per neuron was then calculated. The drebrin immunostaining and rhodamine-phalloidin signal intensity values were used to calculate the drebrin and F-actin SDRs, respectively.

### Statistical analysis

The statistical analysis included a one-way ANOVA followed by a post hoc Scheffe's test. A Student's t test was performed for comparisons between control and drug-treated neurons. All data were presented as mean ± s.e.m. A p value of <0.01 was considered significant. The statistical analysis was performed using Microsoft Excel (Redmond, WA, USA).

## Results

### Quantitative assessment of DA-actin in dendritic spines

We used the spine-dendrite ratio (SDR) of drebrin immunostaining intensity to monitor the amounts of DA-actin in dendritic spines. We used the SDR of rhodamine-phalloidin staining intensity to assess the amount of total F-actin. The SDR used in this study is the average fluorescence signal of the molecule of interest in a dendritic spine head divided by the average fluorescence signal of that molecule in the dendritic shaft at the foot of the spine (Fig. 1A). The drebrin SDR of control cultured hippocampal neurons at 21 days *in vitro* (DIV) was 1.65±0.03 (n = 170 cells), whereas the actin SDR was 1.67±0.02 (n = 170 cells; Fig. 1B), demonstrating that both DA-actin and total F-actin accumulate more in the dendritic spines than the parent dendrites.

### Chemical LTP stimulation induces a transient DA-actin exodus

**Figure 2:**
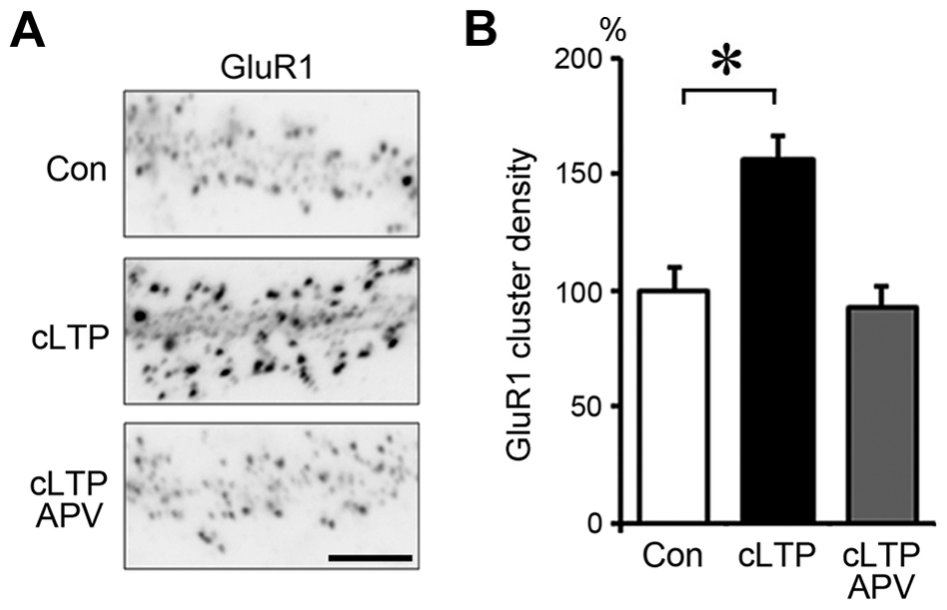
Increase in surface GluR1 immunostaining after chemical LTP (cLTP) stimulation. Neurons (21 DIV) were stimulated with buffer containing 0 µM Mg^2+^, 200 µM glycine, 20 µM bicuculline, 1 µM strychnine and 0.5 µM TTX (cLTP stimulation) for 3 min. (**A**) Surface GluR1 was labeled before the stimulation (top panel; Con) or 30 min after the stimulation (middle panel; cLTP). Note that cLTP stimulation remarkably increased surface GluR1 immunostaining. The increase was completely blocked by APV (bottom panel; cLTP APV). Scale bar, 7 µm. (**B**) Quantitative analysis of surface GluR1 cluster density along dendrites. Data are expressed as percentages relative to the average of control neurons. In the absence of APV, cLTP stimulation significantly increased the density of surface GluR1 clusters (n = 21 cells; p<0.01, Scheffe's test). In contrast, in the presence of APV, no increase in surface GluR1 cluster density was observed following cLTP stimulation (cLTP APV). Error bars represent s.e.m.

Stimulation with chemical LTP (cLTP) solution for 3 min induced a significant increase in the glutamate receptor subunit 1 (GluR1) cluster density 30 min after treatment (Fig. 2). This increase was inhibited with 50 µM 2-amino-5-phosphonopentanoate (APV), an NMDA receptor blocker (Fig. 2). These data are consistent with an earlier study showing facilitated insertion of α-amino-3-hydroxy-5-methyl-4-isoxazolepropionic acid (AMPA) receptors during cLTP [22].

**Figure 3:**
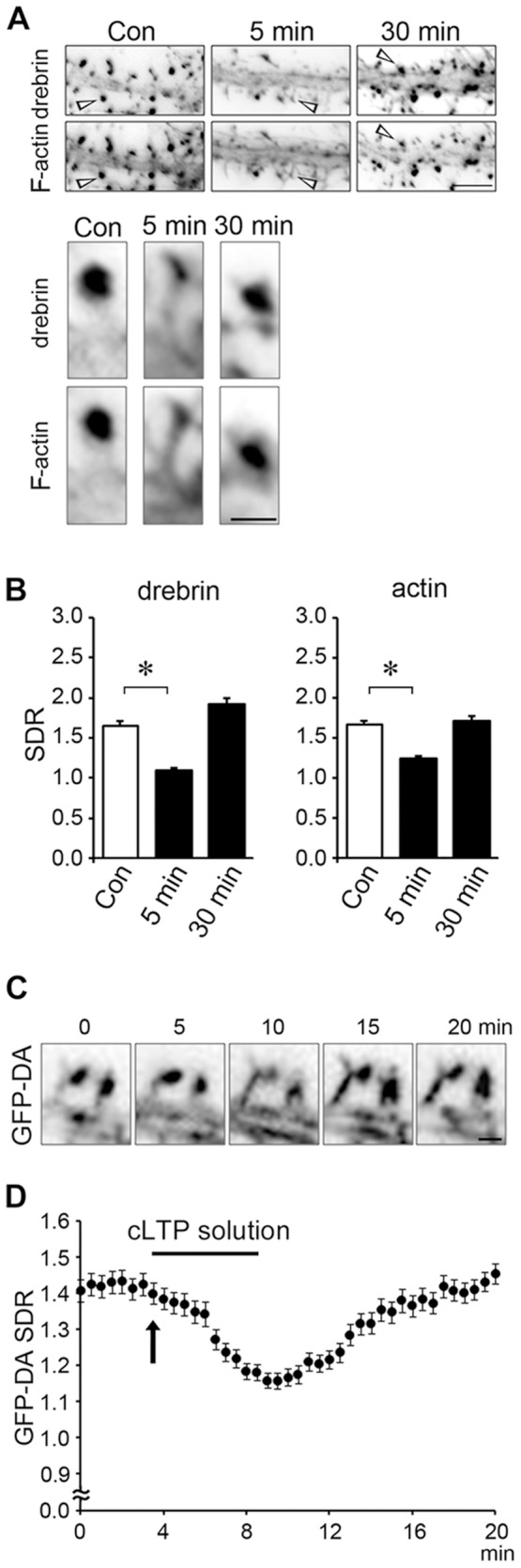
Chemical LTP (cLTP) stimulation induces the exodus and re-entry of DA-actin. (**A**) Neurons (21 DIV) were stimulated with a buffer containing 0 µM Mg^2+^, 200 µM glycine, 20 µM bicuculline, 1 µM strychnine and 0.5 µM TTX (cLTP stimulation) for 3 min and then fixed 5 or 30 min after stimulation. The fixed cells were double-labeled for drebrin and F-actin. Scale bars, 5 µm and 1 µm in upper and lower panels, respectively. The lower panels show the higher-magnification images of the spines (indicated by arrow heads) in the upper panels. (**B**) Bar graphs represent the spine-dendrite ratios (SDRs) for drebrin and actin. cLTP stimulation significantly decreased the drebrin and actin SDRs at 5 min (n = 30 cells; p<0.01, Student's *t* test). Error bars represent s.e.m. (**C, D**) We transfected 7-DIV neurons with a GFP-drebrin A (GFP-DA)-expressing vector and performed time-lapse imaging at 21 DIV. Scale bar, 1 µm. (C) shows the GFP-DA images at 0, 5, 10, 15 and 20 min after the start of the time-lapse recording. The neurons were stimulated with cLTP solution from 3.5 min (indicated by an arrow) to 8.5 min. In (D), closed circles represent data obtained at 30-sec intervals. Error bars represent s.e.m. (n = 7 neurons). The GFP-DA SDR began to decrease soon after cLTP stimulation. When the stimulation was stopped, the GFP-DA SDR began to increase and recovered to control levels within 10 min.

We examined whether cLTP stimulation would induce DA-actin to exit dendritic spines. At 5 min after cLTP stimulation, the intensity of drebrin immunostaining in dendritic spines was weak, but after 30 min the intensity was similar to that in untreated cells (Fig. 3A). Quantitative analysis showed that both drebrin and actin SDRs were significantly lower at 5 min but recovered after 30 min (Fig. 3B).

To analyze the time course of the DA-actin migration, we transfected a GFP-drebrin A-expressing vector into cultured hippocampal neurons and performed time-lapse imaging. The GFP-drebrin A SDR was transiently decreased following cLTP stimulation, similar to that of the native drebrin A (Fig. 3C). The GFP-drebrin A SDR began to decrease immediately after the neurons were exposed to cLTP solution (arrow in Fig. 3D) and then further declined throughout the period of stimulation. When the stimulation medium was switched back to control medium, the SDR began to rise and returned to the control level in 11 min after the completion of cLTP stimulation (Fig. 3D).

### cLTP-induced DA-actin exodus is dependent on myosin II activity

**Figure 4:**
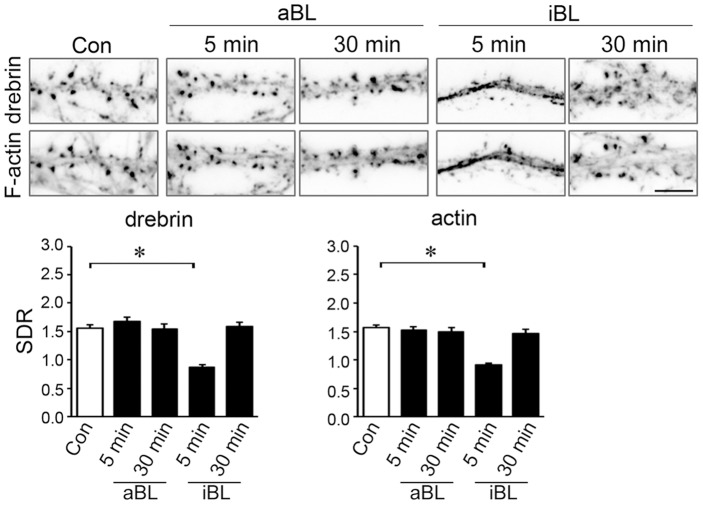
cLTP-induced DA-actin exodus is blocked by an inhibitor of myosin II ATPase. Neurons (21 DIV) were preincubated with 100 µM (S)-(−)-blebbistatin (aBL, the active form of blebbistatin) for 30 min and then stimulated with cLTP solution for 3 min. (R)-(+)-blebbistatin (iBL, the inactive form of blebbistatin) was used as a control. Scale bars, 5 µm. F-actin images indicate that spines kept their structure during the experiment although their shapes were changed. The aBL-treated neurons did not show a decrease in the drebrin and actin SDRs at either 5 min or 30 min after cLTP stimulation (n = 30 cells; Student's test), whereas iBL-treated neurons showed a significant decrease in the drebrin and actin SDRs at 5 min (n = 30 cells; p<0.01, Scheffe's test), similar to that observed in control neurons in Fig. 3. Error bars represent s.e.m.

Neurons were pretreated with 100 µM (S)-(−)-blebbistatin (aBL, the active form of blebbistatin), a myosin II ATPase blocker, for 30 min [30]. In the presence of aBL, cLTP stimulation did not affect the localization of either drebrin or actin in dendritic spines (photomicrographs in Fig. 4), and failed to decrease the drebrin and actin SDRs (n = 30 cells; p = 0.37 for drebrin SDR at 5 min, p = 0.34 for actin SDR; Student's *t* test; graphs in Fig. 4). This result indicates that myosin II activity is involved in the DA-actin exodus. The inactive form of blebbistatin (iBL) was used as a control. In the presence of iBL, cLTP stimulation significantly decreased the drebrin and actin SDRs.

### Various kinds of stimulation induce DA-actin exodus

**Figure 5:**
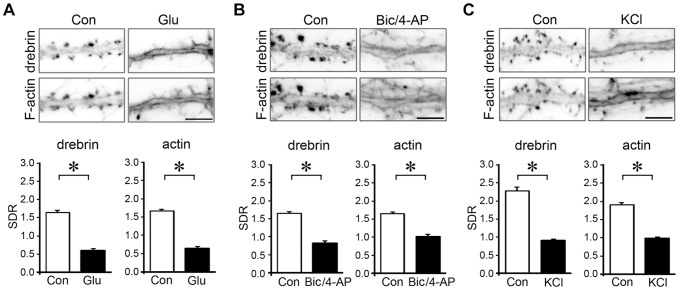
Effects of various excitatory stimulations on DA-actin distribution. Images were obtained from neurons (21 DIV) double-labeled for drebrin and F-actin. Bar graphs represent the spine-dendrite ratios (SDRs) for drebrin and actin. (**A–C**) Neurons were stimulated with 100 µM glutamate for 10 min (A), 50 µM bicuculline and 500 µM 4-aminopyridine (Bic/4-AP) for 10 min (B), or 90 mM KCl in Tyrode's solution for 5 min (C). F-actin images indicate that spines kept their structure during the experiment although their shapes were changed. After stimulation, the drebrin and F-actin clusters in the spines disappeared, and a linear staining pattern appeared along the dendrite. Both the drebrin and actin SDRs were significantly decreased (glutamate, n = 170 cells; Bic/4-AP, n = 30 cells; KCl, n = 30 cells; p<0.01, Student's *t* test). Note that the control drebrin and actin SDRs in (C) were greater than the other SDRs because Tyrode's solution was used instead of normal medium. Scale bars, 5 µm. Error bars represent s.e.m.

We stimulated cultured hippocampal neurons with 100 µM glutamate for 10 min, and fixed them immediately after the stimulation. This treatment induced the loss of drebrin and F-actin from dendritic spines (photomicrographs in Fig. 5A). On the other hand, it did not affect the density of spines or presynaptic terminals (Fig. S1). Quantitative analyses showed significant reductions in both the drebrin and actin SDRs (graphs in Fig. 5A).

We then examined whether other kinds of excitatory stimulation affect DA-actin localization. Increased spontaneous firing rates resulting from a 30-minute application of 50 µM bicuculline, a GABA_A_ receptor blocker, combined with 500 µM 4-aminopyridine, a potassium channel blocker [31], induced a loss of drebrin and F-actin from dendritic spines (photomicrographs in Fig. 5B). A similar decrease was induced by membrane depolarization resulting from a 5-minute application of 90 mM KCl (photomicrographs in Fig. 5C). Quantitative analyses showed that both treatments significantly decreased the drebrin and actin SDRs (graphs in Fig. 5B, C). These data indicate that in addition to cLTP stimulation, various kinds of excitatory stimulation induce a DA-actin exodus.

### Localization of DA-actin and non-DA-actin is differentially regulated by glutamate receptor subtypes

**Figure 6:**
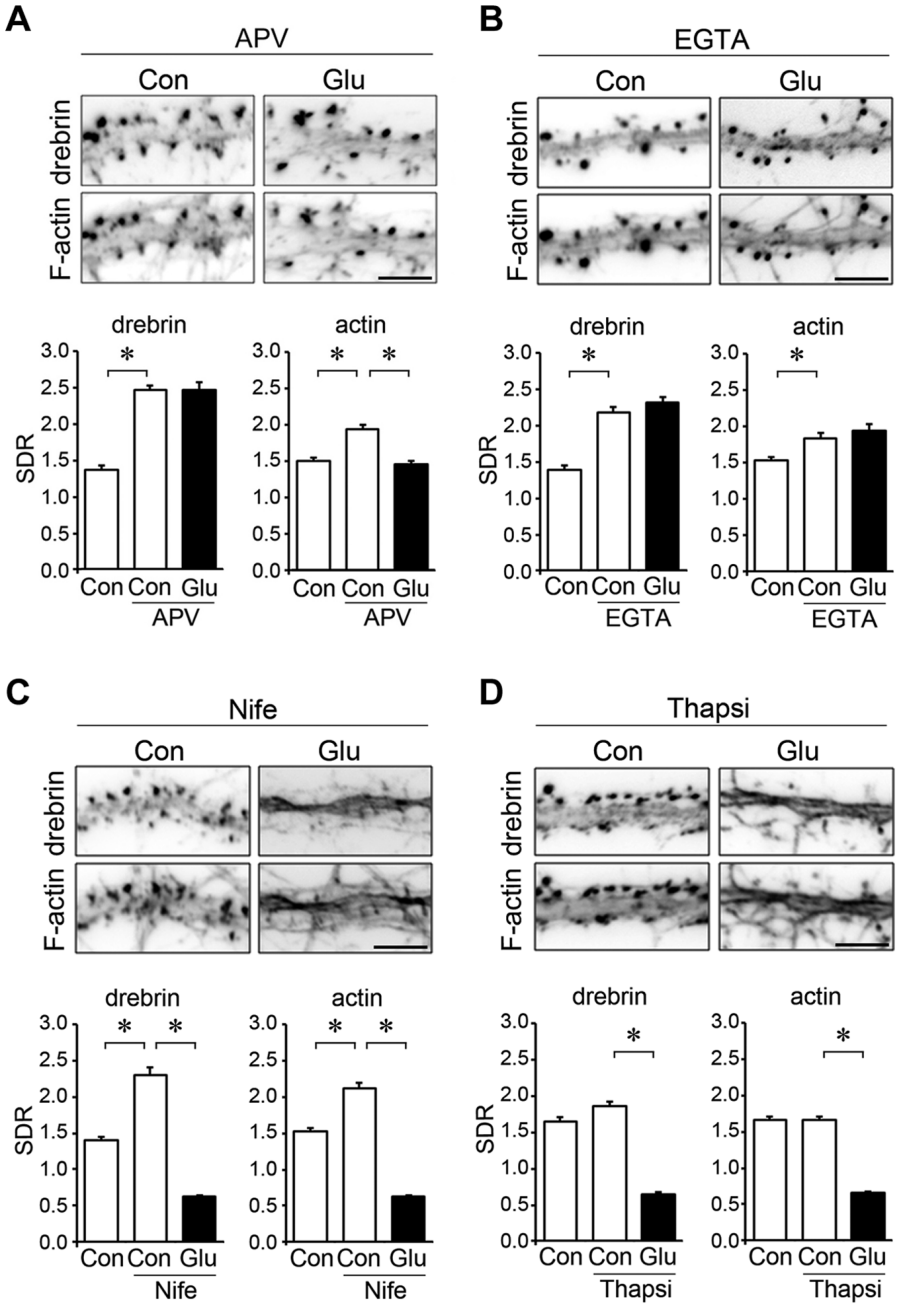
Effects of various inhibitors of Ca^2+^ entry on DA-actin distribution. Neurons (21 DIV) were incubated in normal medium containing 50 µM APV (A), 20 mM EGTA (B), 20 µM nifedipine (C), or 1 µM thapsigargin (D) for 30 min. The neurons were then stimulated with 100 µM glutamate for an additional 10 min. F-actin images indicate that spines kept their structure during the experiment although their shapes were changed. Scale bars, 5 µm. (**A**) APV pretreatment significantly increased both the drebrin and actin SDRs (n = 30 cells; p<0.01, Scheffe's test). In the presence of APV, glutamate stimulation significantly decreased the actin SDR (n = 30 cells; p<0.01, Student's *t* test) but not the drebrin SDR (n = 30 cells; p = 0.52, Student's *t* test). (**B**) EGTA significantly increased the drebrin and actin SDRs (n = 30 cells; p<0.01, Scheffe's test), and blocked the glutamate-induced decreases in drebrin and actin SDRs (n = 30 cells; Student's *t* test). (**C**) Nifedipine significantly increased the drebrin and actin SDRs (n = 30; p<0.01, Scheffe's test), but did not block the glutamate-induced decrease in drebrin and actin SDRs (n = 30; p<0.01, Scheffe's test). (**D**) Thapsigargin neither increased the drebrin and actin SDRs (n = 30; Student's *t* test) nor blocked the glutamate-induced decreases in drebrin and actin SDRs (n = 30; p<0.01, Scheffe's test). Error bars represent s.e.m.

We examined the effect of APV on DA-actin and total F-actin levels in dendritic spines. In the presence of 50 µM APV, drebrin and F-actin were localized at dendritic spines, and this localization was not changed by glutamate stimulation (photomicrographs in Fig. 6A).

However, quantitative analysis showed that the treatment of neurons with APV for 30 min resulted in significant increases in both drebrin and actin SDRs relative to controls. Interestingly, the increase in actin SDR (ca. 130% of control) is smaller than that of the drebrin SDR (ca. 180% of control; Fig. 6A). This indicates that NMDA receptor activity affects the basal accumulation level of DA-actin in dendritic spines more strongly than that of non-DA-actin. If non-DA-actin is not at all affected by APV, the above data suggest that about 40% of total F-actin is DA-actin. Furthermore, in the presence of APV, glutamate stimulation failed to decrease the drebrin SDR but did decrease the actin SDR (Fig. 6A). The disparity between drebrin and actin SDRs indicates that glutamate stimulation decreases the non-DA-actin even in the presence of APV.

Together, it is suggested that the NMDA receptor mediates both the glutamate-induced DA-actin exodus and the basal accumulation of DA-actin in dendritic spines, whereas glutamate receptor subtypes other than the NMDA receptor, such as AMPA or metabotropic glutamate receptors, mediate the non-DA-actin exodus.

### The DA-actin exodus is not regulated by voltage-dependent Ca^2+^ channels or intracellular Ca^2+^ stores

Because NMDA receptor activation leads to Ca^2+^ influx, we examined whether Ca^2+^ regulates the DA-actin distribution. When extracellular Ca^2+^ was chelated by 20 mM ethylene glycol tetraacetic acid (EGTA), the localization pattern of drebrin and F-actin in dendritic spines was similar for both with and without glutamate stimulation (photomicrographs in Fig. 6B). However, quantitative analysis showed that EGTA treatment significantly increased both the drebrin and actin SDRs compared with control neurons. Following the extracellular Ca^2+^ chelation, glutamate stimulation failed to induce decreases in drebrin and actin SDRs (n = 30 cells; p = 0.88 for drebrin SDR, p = 0.84 for actin SDR; Student's *t* test; graphs in Fig. 6B). These data indicate that Ca^2+^ influx is involved in the changes in both DA-actin and non-DA-actin distribution.

Inhibition of L-type voltage-dependent Ca^2+^ channels with 20 µM nifedipine did not block glutamate-induced changes in drebrin and F-actin localization (photomicrographs in Fig. 6C). Quantitative analysis also showed that nifedipine treatment did not inhibit the glutamate-induced decreases in drebrin and actin SDRs. However, in the absence of glutamate stimulation, nifedipine treatment significantly increased the drebrin and actin SDR levels, similar to the results obtained with APV and EGTA treatments (graphs in Fig. 6C). This indicates that voltage-dependent Ca^2+^ channels regulate the accumulation of DA-actin in dendritic spines, but do not regulate the DA-actin exodus. However, we cannot exclude the possibility that the increase of the basal SDR is due to inhibition of voltage-dependent Ca^2+^ channels in the presynaptic terminus.

Inhibition of Ca^2+^ release from intracellular stores with 1 µM thapsigargin [32] did not block glutamate-induced changes in drebrin and F-actin localization (photomicrographs in Fig. 6D). Quantitative analysis showed that thapsigargin neither increased the drebrin and actin SDRs (n = 30 cells; p = 0.99 for drebrin SDR, p = 0.50 for actin SDR; Student's *t* test) nor blocked the glutamate-induced decreases in drebrin and actin SDRs (n = 30 cells: p<0.01, Scheffe's test; graphs in Fig. 6D).

Together, these data indicate that DA-actin exodus is regulated by NMDA receptors, but not by voltage-dependent Ca^2+^ channels. On the other hand, the basal accumulation of DA-actin in dendritic spines is regulated by both NMDA receptors and voltage-dependent Ca^2+^ channels. Ca^2+^ release from intracellular stores is not involved in either the DA-actin exodus or the basal accumulation of DA-actin.

### Glutamate-induced DA-actin exodus is also dependent on myosin II ATPase activity

**Figure 7:**
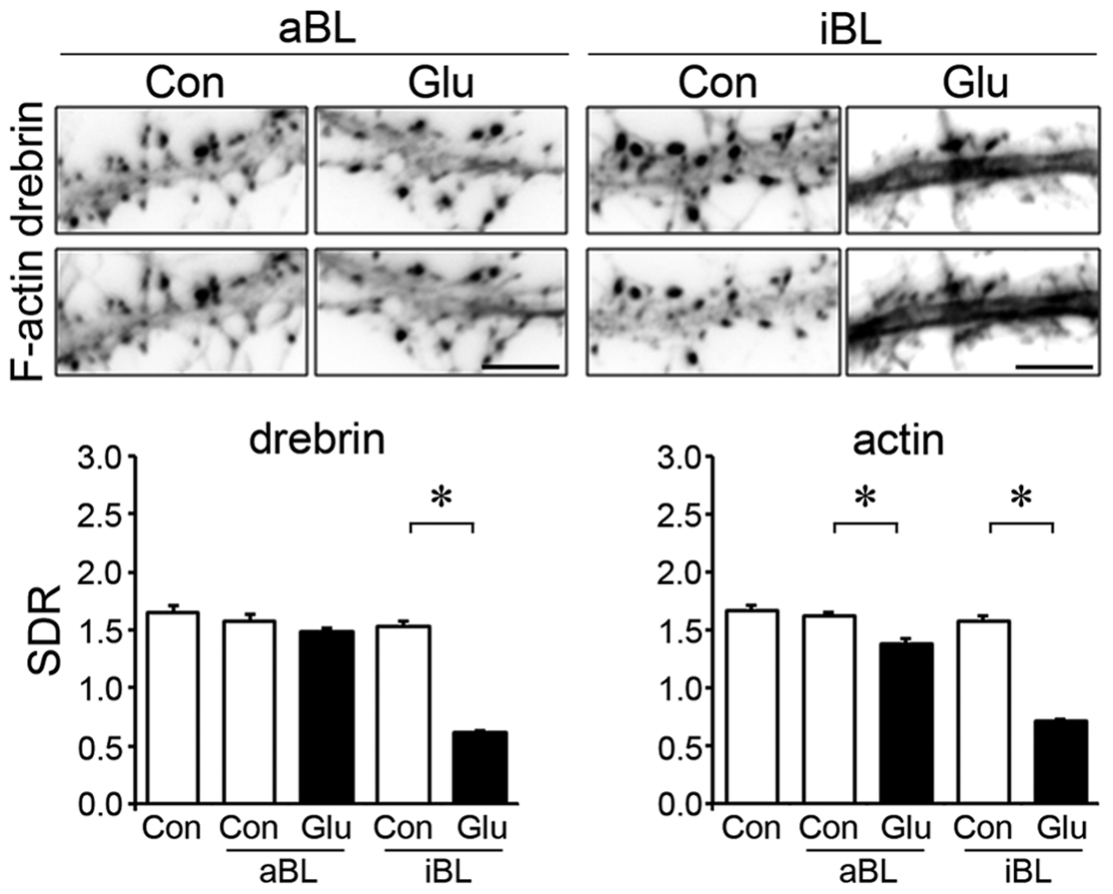
Glutamate-induced DA-actin exodus is blocked by an inhibitor of myosin II ATPase. Neurons (21 DIV) were preincubated with 100 µM aBL for 30 min and then stimulated with 100 µM glutamate for 10 min. F-actin images indicate that spines kept their structure during the experiment although their shapes were changed. Scale bars, 5 µm. The drebrin SDR of aBL-treated neurons was not decreased by glutamate stimulation (n = 30 cells; p = 0.06, Student's *t* test), although that of iBL-treated neurons was decreased (n = 30 cells: p<0.01, Scheffe's test). On the other hand, the actin SDR of aBL-pretreated neurons was slightly, but significantly, decreased by glutamate stimulation (n = 30 cells; p<0.01, Scheffe's test), although the reduction was much smaller than that observed in iBL-pretreated neurons (n = 30 cells; p<0.01, Student's *t* test). Error bars represent s.e.m.

We examined whether myosin II ATPase is involved in the glutamate-induced DA-actin exodus. In the presence of aBL, drebrin localization at dendritic spines was not affected by glutamate stimulation (photomicrographs in Fig. 7), and glutamate stimulation did not induce a decrease in drebrin SDR (n = 30 cells; p = 0.06, Student's *t* test; graph in Fig. 7). Interestingly, the actin SDR was slightly, but significantly, decreased upon glutamate stimulation (graph in Fig. 7), although the decrease was not remarkable compared with that in the presence of iBL (Fig. 7). This result indicates that inhibition of myosin II ATPase does not completely block the exodus of F-actin, indicating that a small amount of F-actin other than DA-actin exits dendritic spines in response to glutamate stimulation.

Together, it is indicated that the glutamate-induced as well as the cLTP-induced DA-actin exodus depends on myosin II ATPase, but the glutamate-induced non-DA-actin exodus is at least partly independent of myosin II ATPase. This myosin II-independent loss of non-DA-actin might correspond to the NMDA receptor-independent loss of non-DA-actin shown in Fig. 6A.

### The DA-actin exodus is not dependent on phosphorylation of myosin light chain

**Figure 8:**
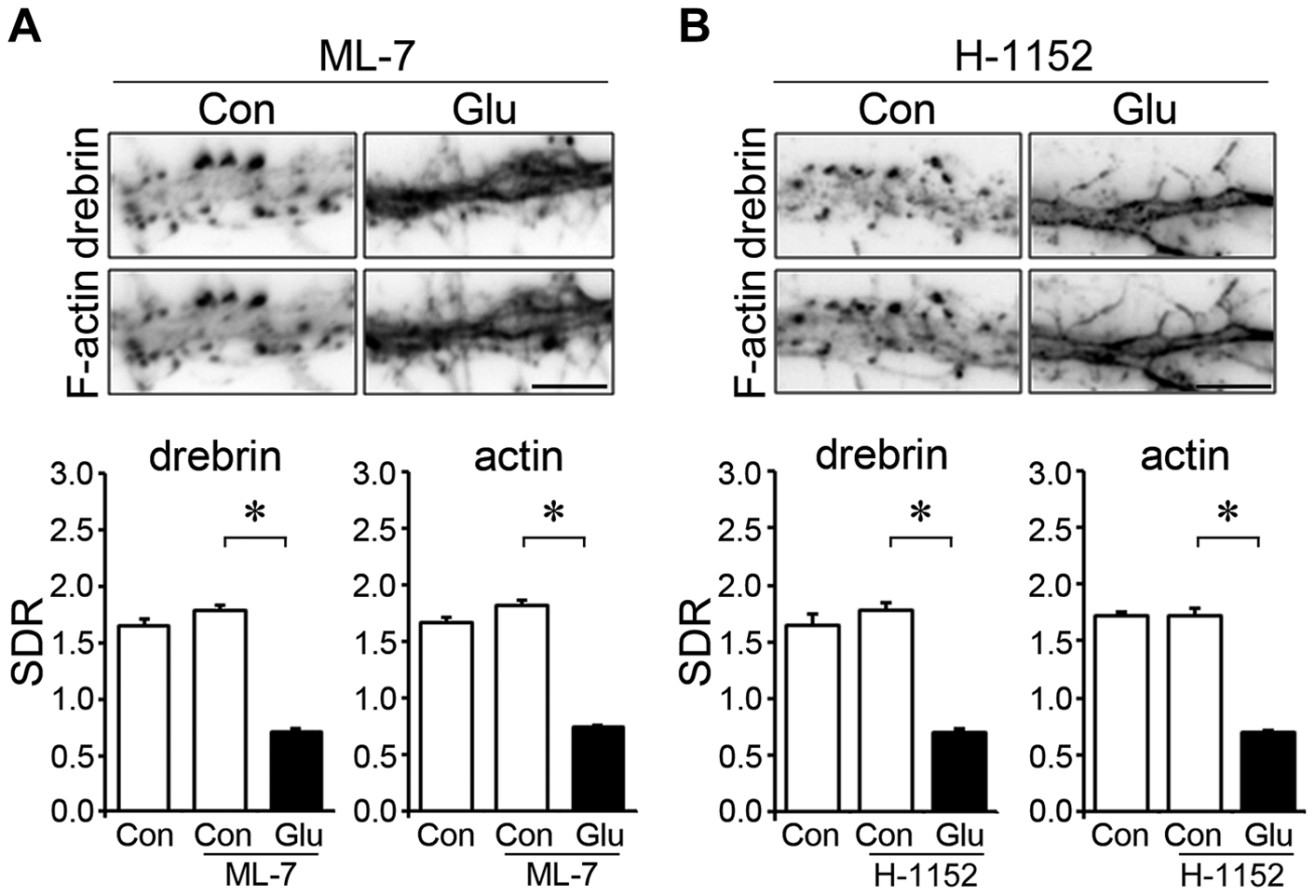
The DA-actin exodus is not blocked by inhibitors of myosin light chain kinase (MLCK) or Rho-associated kinase (ROCK). Neurons (21 DIV) were preincubated with 10 µM of ML-7, an inhibitor of MLCK (A) or 1 µM H-1152, an inhibitor of ROCK (B) for 30 min, and then stimulated with 100 µM glutamate for 10 min. Neither ML-7 (n = 30 cells; drebrin SDR p<0.01, actin SDR p<0.01, Scheffe's test) nor H1152 (n = 30 cells; drebrin SDR p<0.01, actin SDR p<0.01, Scheffe's test) blocked the DA-actin exodus. F-actin images indicate that spines kept their structure during the experiment although their shapes were changed. Scale bars, 5 µm.

To examine whether the phosphorylation of myosin light chain (MLC) is involved in the DA-actin exodus, we inhibited myosin light chain kinase (MLCK). When MLCK activity was inhibited with 10 µM ML-7, glutamate stimulation induced the loss of drebrin and F-actin from dendritic spines (photomicrographs in Fig. 8A). Quantitative analysis showed that the ML-7 treatment did not change the drebrin and actin SDR levels compared with control neurons, and did not inhibit the glutamate-induced decreases in the drebrin and actin SDRs (Fig. 8A).

We then inhibited ROCK activity with 1 µM H-1152. In the presence of H-1152, glutamate stimulation induced the loss of drebrin and F-actin from dendritic spines (photomicrographs in Fig. 8B). Quantitative analysis showed that the H-1152 treatment did not change the drebrin and actin SDR levels compared with control neurons, and did not inhibit the glutamate-induced decreases in the drebrin and actin SDRs (Fig. 8B).

Because MLCK and ROCK phosphorylate MLC [33], the above data suggest that MLC phosphorylation is not involved in the DA-actin exodus.

## Discussion

In the present study we demonstrated that (1) chemical long-term potentiation (cLTP) stimulation induces rapid DA-actin exodus and subsequent DA-actin re-entry in dendritic spines, (2) Ca^2+^ influx through NMDA receptors regulates both the exodus and the basal accumulation of DA-actin, and (3) the DA-actin exodus is blocked by a myosin II ATPase inhibitor, but is not blocked by either MLCK or ROCK inhibitors.

These results indicate that Ca^2+^ influx through NMDA receptors induces the DA-actin exodus in LTP induction, and that myosin II mediates the interaction between NMDA receptor activation and DA-actin exodus (Fig. S2). Furthermore, the Ca^2+^ influx seems to activate myosin II ATPase by a rapid actin-linked mechanism instead of slow MLC phosphorylation. Thus the myosin II-mediated DA-actin exodus might be an initial event in LTP induction, triggering actin polymerization and spine enlargement.

### SDR analysis of DA-actin migration in and out of dendritic spines

In the present study, using drebrin SDR, we found that APV treatment not only inhibits the DA-actin exodus but also facilitates the accumulation of DA-actin in dendritic spines. In our previous studies, we could not detect any facilitative effect of APV treatment on drebrin accumulation in dendritic spines, because we used drebrin cluster density along dendrites for assessing the dynamic changes in drebrin localization [13], [17]. Although this method is sensitive enough to detect the loss of drebrin from dendritic spines, it is not sensitive enough to detect the accumulation of drebrin in dendritic spines.

By comparing the changes in drebrin and actin SDRs we can extrapolate the changes in non-DA-actin, because the total F-actin shown by the actin SDR consists of DA-actin and non-DA-actin. In the present study we found that the glutamate-induced exoduses of DA-actin and non-DA-actin are differentially regulated by each glutamate receptor subtype. Thus measurement of SDR is a useful method to analyze the migration of proteins in and out of dendritic spines.

### The DA-actin exodus may trigger the facilitation of F-actin polymerization in dendritic spines

After NMDA receptor activation in LTP induction, facilitation of actin polymerization and spine enlargement are observed [34]–[35]. However, the underlying mechanisms of these processes have not been elucidated. The present study shows that the total amount of F-actin in dendritic spines transiently decreases shortly after cLTP stimulation, resulting from a DA-actin exodus. Once the total F-actin in dendritic spines reduces by the DA-actin exodus, monomeric actin is likely to immediately refill the vacant space by diffusion [15]. The increase in the amount of monomeric actin is known to facilitate F-actin polymerization [36]. In addition, the treadmilling rate of DA-actin is low [8], suggesting that the high level of DA-actin in dendritic spines makes the average treadmilling rate of total F-actin lower in resting dendritic spines. Because quickly-treadmilling non-DA-actin predominates in dendritic spines after the DA-actin exodus, the average treadmilling rate of total F-actin is increased. Together, it is indicated that the DA-actin exodus increases the monomeric actin content and the treadmilling rate of F-actin in dendritic spines, resulting in the facilitation of F-actin polymerization and spine enlargement (Fig. S2).

### Possible molecular mechanism for how Ca^2+^ influx activates myosin II ATPase in dendritic spines

Myosin II ATPase is known to be activated by MLC phosphorylation or by an actin-linked mechanism. The important issue remaining is which molecular mechanism is related to the DA-actin exodus. MLCK and ROCK are two major candidates for the regulator of MLC phosphorylation [33]. The present study reveals that block of neither MLCK nor ROCK inhibits the DA-actin exodus. This finding suggests that MLC phosphorylation is not involved in the DA-actin exodus.

Thus activation of myosin II ATPase by an actin-linked mechanism is likely involved in the DA-actin exodus. In the actin-linked mechanism, myosin II ATPase is activated by the release of the suppressed actomyosin interaction. In mammalian skeletal muscles, when Ca^2+^ binds to the troponin complex, the actomyosin interaction suppressed by tropomyosin is released, and consequently myosin II ATPase is activated. Because drebrin A inhibits the myosin II ATPase activity similar to tropomyosin [20], drebrin A is thought to be the counterpart of tropomyosin in dendritic spines [14]. Therefore it is suggested that drebrin A protects actin filaments from the interaction with myosin II in the resting dendritic spines, resulting in the inhibition of myosin II ATPase. Once NMDA receptors are activated, the Ca^2+^ influx through NMDA receptors may change the location of drebrin A on the actin filaments, releasing the actomyosin interaction suppressed by drebrin A. Consequently myosin II ATPase is activated in dendritic spines. Thus myosin II ATPase activation by an actin-linked mechanism may be an underlying molecular mechanism for the DA-actin exodus.

Furthermore, the present data shows that the DA-actin exodus occurs immediately after cLTP stimulation. The activation of myosin II ATPase by an actin-linked mechanism occurs within 20 ms after stimulation [37]. Thus the myosin-II mediated DA-actin exodus might be an initial event in LTP induction, triggering actin polymerization and spine enlargement.

### Role of DA-actin re-entry into dendritic spines

DA-actin re-entry follows the DA-actin exodus. In the present study we found that the LTP-induced DA-actin exodus triggers F-actin polymerization and spine enlargement. The DA-actin re-entry may be related to maintenance of LTP. After the DA-actin re-entry, the dynamic and stable F-actin pools are probably reestablished in the dendritic spines. As a result, polymerization and depolymerization of F-actin is balanced in dendritic spines and the enlarged spine morphology is maintained (Fig. S2). In fact, long lasting increases in F-actin and drebrin content in dendritic spines have been reported when LTP is maintained *in vivo* [38].

What are the underlying mechanisms of DA-actin re-entry? Reduction of the Ca^2+^ influx into dendritic spines might induce the DA-actin re-entry, because the basal accumulation of DA-actin is negatively regulated by Ca^2+^ influx through NMDA receptors and voltage-dependent Ca^2+^ channels in resting spines. Another possible mechanism is a signaling cascade linked to AMPA receptors. LTP stimulation is known to increase the AMPA receptor density in dendritic spines [39]. Because AMPA receptor activity facilitates accumulation of DA-actin in the dendritic spines of immature neurons [17], AMPA receptors might also be involved in the DA-actin re-entry in mature neurons.

## Supporting Information

Figure S1 **Effects of glutamate stimulation on the density of spines and presynaptic terminals.** (**A**) DiI staining of hippocampal neurons. Fixed cultures on coverslips were bathed in PBS and placed on the stage of an inverted phase microscope. Individual cells were stained with 1,1′-dioctadecyl-3,3,3′,3′-tetramethylindocarbocyanine perchlorate (DiI; Molecular Probes, Inc., Eugene, OR). The DiI was dissolved in vegetable oil to saturation, loaded into a glass micropipette (Eppendorf), and applied by pressure ejection onto the multipolar neurons. The coverslips were then placed at room temperature in small petri dishes containing PBS. After 12–24 h, which allowed for sufficient transport of the dye, cells were examined by fluorescence microscopy. The spine morphology of boxed areas in upper panels are shown at higher magnification in middle panels. Note that the spines kept their structures during the experiment. Scale bars, 10 µm. Left panel in the bottom shows the density of dendritic spines. To measure spine density, the number of spines per cell was then counted for 25 cells (50–100 µm total dendritic length per neuron). Significant differences were not observed between the control (n = 42 dendrites) and glutamate-treated dendrites (n = 44 dendrites; p = 0.73, Student's t test). Bar graphs represent dendritic spine density. The cumulative frequency plots in the bottom show distribution of spine length and spine width. The glutamate treatment significantly increased the spine length (control, 1.39±0.03 µm, n = 42 dendrites; glutamate, 1.72±0.05 µm, n = 44 dendrites, p<0.01, Student's t test) and reduced the spine width (control, 0.96±0.01 µm, n = 42 dendrites; glutamate, 0.82±0.02 µm, n = 44 dendrites, p<0.01, Student's t test). (**B**) Triple-labeled images of drebrin, F-actin and synapsin I in hippocampal neurons. Scale bars, 10 µm. (**C**) Bar graphs represent the density of synapsin I clusters. Synapsin I cluster density was measured according to previously described methods (Takahashi et al., 2009). No significant differences in the density of synapsin I clusters were detected between control (n = 30 dendrites) and glutamate-stimulated neurons (n = 30 dendrites; p = 0.86, Student's t test). Data are presented as mean ± s.e.m.
(TIF)

Figure S2 **Model for architectural changes in the actin cytoskeleton during LTP formation.** (**A**) Dendritic spines in the resting state contain a dynamic F-actin pool (non-DA-actin) at the tip of the spine head, and a stable pool (DA-actin) in the base of the spine head. Although the dynamic F-actin pool shows quick treadmilling, polymerization and depolymerization of F-actin is balanced, consequently maintaining spine morphology. (**B**) Once Ca^2+^ enters through NMDA receptors, it activates myosin II ATPase through disinhibition of the DA-actin and myosin-II interaction. Consequently DA-actin exits the dendritic spine head and simultaneously monomeric actin refills the vacant space in the spine head. Both these changes cooperate to facilitate the polymerization of non-DA-actin, which is the predominant component of an enlargement F-actin pool in the spine head. Accordingly the spine head is enlarged. (**C**) Ca^2+^ reduction and/or APMA receptor activation induce DA-actin re-entry. The DA-actin re-entry reconstitutes the dynamic and stable F-actin pools in dendritic spines, contributing to maintenance of the enlarged spine morphology until the next DA-actin exodus is triggered.
(EPS)
