# Supplementary figures and images for: Myosin II ATPase Activity Mediates the Long-Term Potentiation-Induced Exodus of Stable F-Actin Bound by Drebrin A from Dendritic Spines
Source: PLoS One. 2014 Jan 22;9(1):e85367. doi: 10.1371/journal.pone.0085367 (PMC3899004; doi:10.1371/journal.pone.0085367)

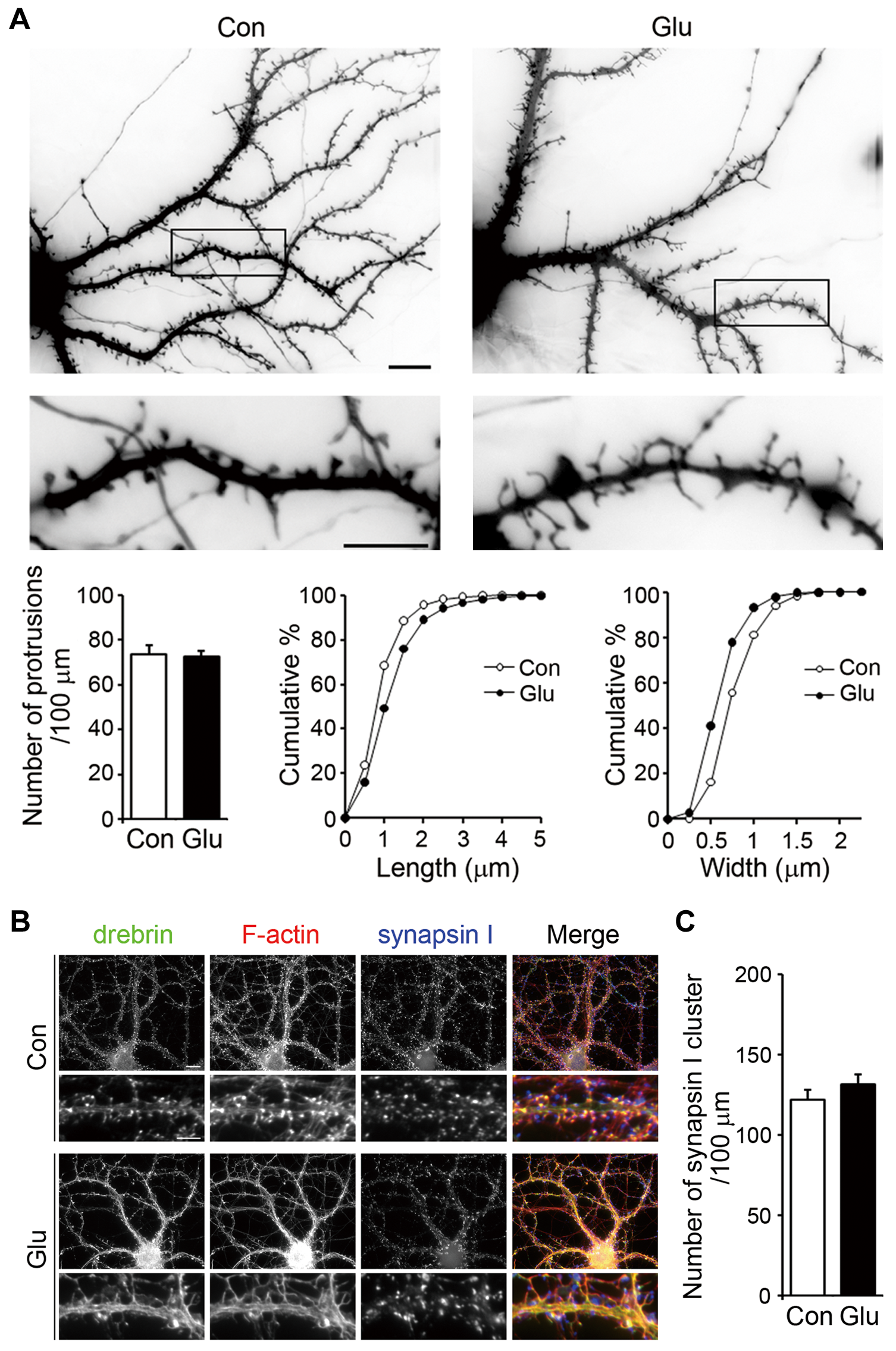

Supplement: Figure S1 — Effects of glutamate stimulation on the density of spines and presynaptic terminals. (A) DiI staining of hippocampal neurons. Fixed cultures on coverslips were bathed in PBS and placed on the stage of an inverted phase microscope. Individual cells were stained with 1,1′-dioctadecyl-3,3,3′,3′-tetramethylindocarbocyanine perchlorate (DiI; Molecular Probes, Inc., Eugene, OR). The DiI was dissolved in vegetable oil to saturation, loaded into a glass micropipette (Eppendorf), and applied by pressure ejection onto the multipolar neurons. The coverslips were then placed at room temperature in small petri dishes containing PBS. After 12–24 h, which allowed for sufficient transport of the dye, cells were examined by fluorescence microscopy. The spine morphology of boxed areas in upper panels are shown at higher magnification in middle panels. Note that the spines kept their structures during the experiment. Scale bars, 10 µm. Left panel in the bottom shows the density of dendritic spines. To measure spine density, the number of spines per cell was then counted for 25 cells (50–100 µm total dendritic length per neuron). Significant differences were not observed between the control (n = 42 dendrites) and glutamate-treated dendrites (n = 44 dendrites; p = 0.73, Student's t test). Bar graphs represent dendritic spine density. The cumulative frequency plots in the bottom show distribution of spine length and spine width. The glutamate treatment significantly increased the spine length (control, 1.39±0.03 µm, n = 42 dendrites; glutamate, 1.72±0.05 µm, n = 44 dendrites, p<0.01, Student's t test) and reduced the spine width (control, 0.96±0.01 µm, n = 42 dendrites; glutamate, 0.82±0.02 µm, n = 44 dendrites, p<0.01, Student's t test). (B) Triple-labeled images of drebrin, F-actin and synapsin I in hippocampal neurons. Scale bars, 10 µm. (C) Bar graphs represent the density of synapsin I clusters. Synapsin I cluster density was measured according to previously described methods (Takah [file pone.0085367.s001.tif]
